# How to use Twitter at a Scientific Conference

**DOI:** 10.1128/msphere.00121-22

**Published:** 2022-05-09

**Authors:** B. Joanne Power

**Affiliations:** a Institute of Infection, Immunity, and Inflammation, College of Medical, Veterinary and Life Sciences, University of Glasgow, Sir Graeme Davies Building, 120 University Place, Glasgow, United Kingdom; University at Buffalo

**Keywords:** Twitter, communications, outreach, live-tweeting, applications, software, science communication

## Abstract

In the past decade, social media platforms have been recognized as an important tool in the dissemination of science among the research community and as an interface between scientists and the general public. Publishing companies that specialize in scientific research now pay attention to alternative metrics (“altmetrics”) and provide comprehensive guides about social media management to editors. Twitter has emerged as a leader among social media platforms in the dissemination of science. This Perspective will assert the merits of using Twitter to expand the reach of scientific conferences while providing guidance on how to disseminate conference findings in real-time, called “live-tweeting,” without compromising scientific integrity.

## PERSPECTIVE

Upon finding myself jetlagged at a conference in 2016, I attempted to stay focused on the presentations of conference speakers by publishing conference events on Twitter in real-time, a process known as “live-tweeting.” By the end of the first session, comments had started rolling in from other scientists who had been unable to attend the event, and it became apparent to me that I was providing a valuable service. Since then, I have live-tweeted 16 further conferences, stopping only if I had to present my own research. In this Perspective, I hope to impart some of the knowledge I have gained in using Twitter to provide real-time updates for scientific conferences, whether attending in-person or virtually.

Among scientists, Twitter has been recognized as a positive networking tool at all career stages (1, 2), is accessible to audiences in lower- and middle- income countries (LMICs) (3), and can foster accessibility and inclusion in science with the use of image-rich outreach and alternative text (“alt text”) (4). Social media platforms such as Twitter have also been instrumental in the creation of communities of practice (COPs) by facilitating regular contact between users with shared passions or challenges in STEM (science, technology, engineering, and mathematics), such as women, minorities, and LGBTQ+ scientists (5–7).

In medicine, Twitter has been acknowledged as a tool for online discussion at conferences, facilitating engagement between speakers and the audience and allowing those who could not attend in-person to keep abreast of conference presentations and discussions (8, 9). Fewer articles and blogs have been written about how to use Twitter at basic science conferences (10–12). Scientific conferences cater primarily to scientists from resource-rich institutions in developed countries, creating a barrier for scientists from LMICs (13), mothers in academia (14), scientists with disabilities (15), and those in academic science who are also primary caregivers (16). Twitter is another tool in our belt to facilitate open access and equity in science.

## SETTING UP AN ACADEMIC TWITTER ACCOUNT

**FIG 1:**
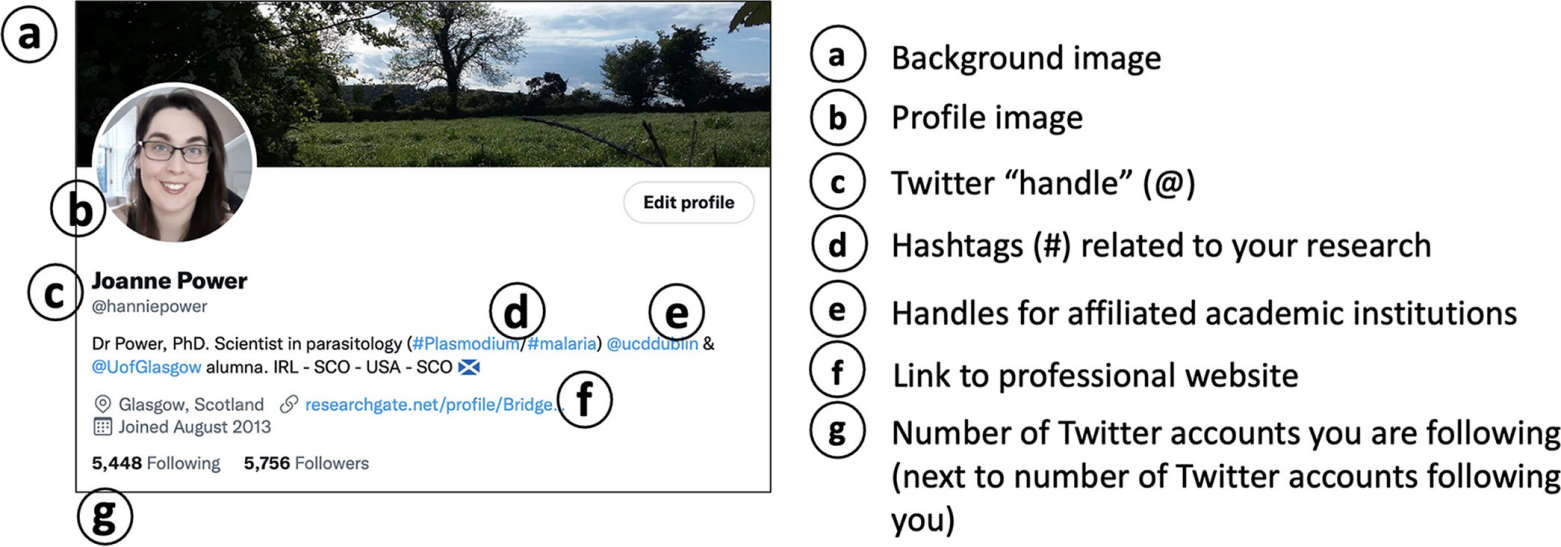
A basic academic Twitter profile. An example of the author’s own Twitter account used for scientific purposes. Profile components are labeled a to g.

The basic components of an academic Twitter account are shown in Fig. 1. Twitter is free of charge and only requires an email address and the creation of a password to register as a user. A Twitter “handle,” the “@” symbol followed by a username, is used to identify an individual on the platform (Fig. 1C). On Twitter, the background image (Fig. 1A) is only seen when a user clicks on a Twitter handle to specifically view a profile page. This background image does not necessarily have to be related to a scientist’s field of study. More important is the profile image (Fig. 1B), which can be seen when the user posts or comments on another tweet. As this image is viewed more, it should be identifiable, for example, a headshot of the individual that can be matched to the individual’s other professional social media accounts, such as LinkedIn or Google Scholar.

Twitter also allows users to add a 160-character description underneath their profile photo and handle. This description can be used to add hashtags (“#” followed by text) that can describe the individual’s scientific interests or areas of research (Fig. 1D). The user can also tag the Twitter handles of the institutes in which they studied or worked (Fig. 1E). A useful addition for a public academic Twitter profile is to use the “website” option when creating the account to enter the URL of a second professional account, such as a laboratory website, ORCID account, or ResearchGate profile (Fig. 1F). Twitter will also show you how many Twitter accounts you follow and those who follow your Twitter account (Fig. 1G).

## LIVE-TWEETING SCIENTIFIC CONFERENCES: A “HOW-TO” AND BEST PRACTICES

**FIG 2:**
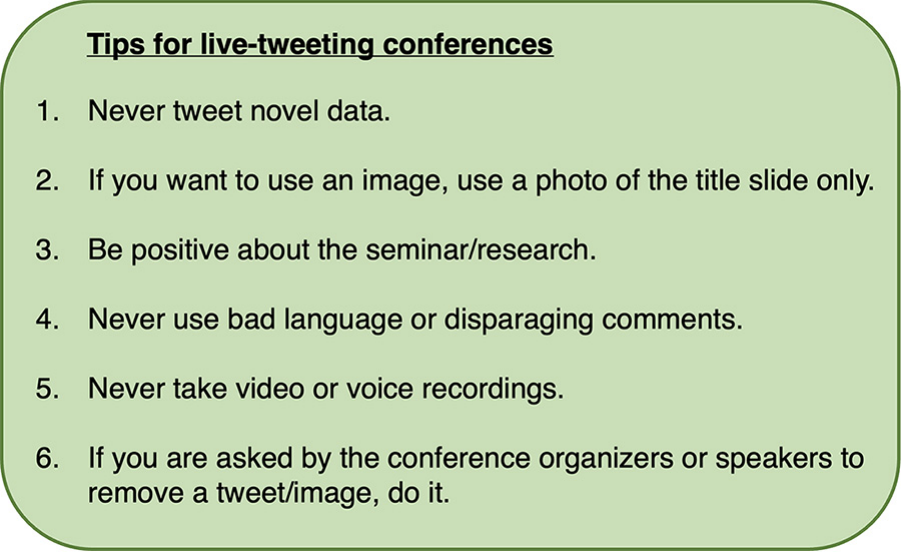
Tips for live-tweeting conferences. Six tips used by the author to maintain scientific integrity when live-tweeting updates about presentations during a scientific conference.

Conferences play an important role in the dissemination of novel scientific research, and Twitter can be used to expand the reach of scientific conferences. At present, scientific conference organizers do not always provide a social media manager to live-tweet events, and the task often falls to members of the conference who are more familiar with Twitter. Figure 2 below provides a few basic, helpful tips to live-tweeting a conference while protecting the intellectual property of the conference speakers.

**FIG 3:**
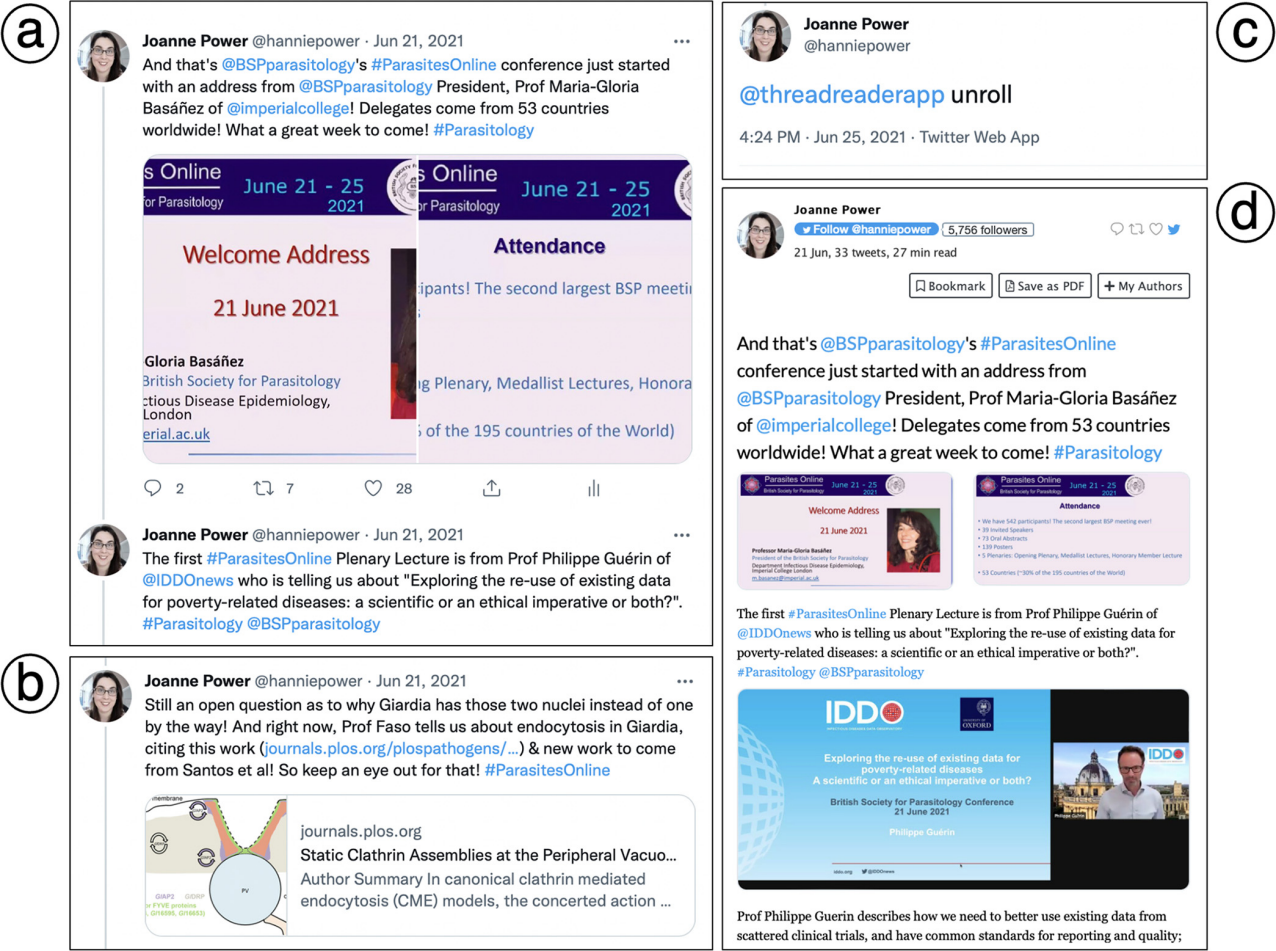
Live-tweeting an academic science conference. This figure provides examples of how to live-tweet a scientific conference using the author’s live-tweeting of the British Society for Parasitology (BSP) “Parasites Online” conference, which was held virtually during the COVID-19 pandemic from 21 to 25 June 2021. (a) An example of how to use tagged Twitter accounts and hashtags to spread awareness of the conference in a Twitter “thread.” (b) An example of how to share previous articles or preprints from a conference speaker in a tweet. (c) An example of the tweet used to generate a new tab using the Thread Reader App, which can be an easier way to read a long Twitter thread at the end of a conference session. (d) An example of a new tab linking all tweets in a thread together in one document as generated using the Thread Reader App.

There are a number of ways to maximize your reach when using Twitter to live-tweet a scientific conference. As shown in Fig. 3A, using the example of a conference that was held virtually from 21 to 25 June 2021 (the British Society for Parasitology [BSP] “Parasites Online” conference), both handles for the official BSP Twitter account and that of the Society President’s institute are tagged (using the “@” symbol). Tagging societies, institutes, and other individuals alerts them to your tweet, which may prompt them to respond and/or broadcast it further with a retweet.

Most conferences will now provide an official conference hashtag, in the example case, it is “#ParasitesOnline” (Fig. 3A). Hashtags can be searched for, meaning that all tweets containing “#ParasitesOnline” can be viewed together, generating a wealth of conference notes and comments from many individuals who have attended, want to ask questions, or discuss findings. Multiple attendees live-tweeting a conference at the same time does not make the activity redundant in any way but merely adds additional points of view to the discourse. In Fig. 3A, the second tweet is added to the first in a “thread,” i.e., a series of tweets that follow one another in sequence. You can add a tweet to another in a thread by clicking on the “reply” symbol on the tweet you want to add to. By keeping all tweets in one thread, people can easily follow the conference along, or go back if they logged in late. Maintaining a thread also enables the use of the Thread Reader App later (Fig. 3C).

A tweet that introduces a conference speaker can be followed with more information as in Fig. 3B. In some cases, the scientific findings/data being presented by the speaker are already available as a peer-reviewed publication or as a preprint. A quick Internet search for the scientist will often lead to a list of their publications, in which case the URL can be copied and pasted into the tweet, where Twitter will automatically generate an image with a title as shown in Fig. 3B. If an issue arises where the URL or multiple URLs exceed the character limit of 280 characters on Twitter, freely-available URL shorteners such as Bitly (http://bitly.ws) or TinyURL (https://tinyurl.com/app) can be used to shorten the links.

In recent years, one of the most useful online tools one can use when live-tweeting conferences has been the Thread Reader App (https://threadreaderapp.com), which can be applied at the end of a thread as shown in Fig. 3C. When the Thread Reader App is mentioned in a reply at the very end of the thread, followed by a space and the word “unroll,” a new tab is opened in which all of the tweets in that particular thread can be viewed outside of the Twitter app in sequence (Fig. 3D). A Thread Reader tab can make the information that has been tweeted easier to read if a thread in Twitter becomes cumbersome. The Thread Reader App is free to use but does offer a subscription service in which Twitter threads can be converted to PDF for easy reading at a later time. For a conference that takes place over a number of days, it is sometimes more useful to create separate Twitter threads for each day and to end each day by summarizing with the Thread Reader App.

## POSTER SESSIONS AND MORE USEFUL APPS

Twitter has become a useful tool for scientists from all fields of research to network, keep abreast of science news, and even boost the number of citations a publication gains long-term (17). Specific Twitter pages have also been created by most publishers, job recruiters, and funding agencies, giving scientists easy access to newly published articles, job opportunities, and funding calls with a simple scroll of their Twitter “feed.” Twitter allows users to send other accounts private, direct messages (“DMs”), which creates opportunities for scientists across the world to engage in private conversation and, potentially, to collaborate on projects. With the creation of an individual account as shown in Fig. 1 and using the tips described in Fig. 2 and 3, anyone can successfully live-tweet a scientific conference while maintaining scientific integrity.

While Fig. 2 and 3 address how to live-tweet presentations/seminars at scientific conferences, tweeting about conference poster sessions is much simpler. A scientific poster is a single representation of a research project, with text that is often too small to be of use when an image is taken. Scientific posters also often contain novel data, and so it is recommended that poster session titles are simply advertised on Twitter but that an image of the actual poster is not shared online. In some cases, a student or postdoctoral researcher may be happy for a photograph of them to be taken alongside their poster. In this case, sharing an image of a scientific poster on Twitter is acceptable but only with the express approval of the poster presenter.

There are additional tools that may become useful if an individual user gains access to multiple Twitter accounts or multiple social media platforms, for example, an individual employed to manage social media platforms for a company or a publishing house. When tasked with managing more than one Twitter account, the free social media dashboard application “TweetDeck” (https://tweetdeck.twitter.com) can allow the user to log into multiple Twitter accounts at once and monitor the content released from each account. TweetDeck can prove particularly useful for learned societies or institutions with social media/communications teams made up of more than one person. Each Twitter account can have one or more administrators with the ability to grant further access to members of a communications team. Similarly, commercial social media marketing and management dashboard applications, such as “Hootsuite” (https://www.hootsuite.com), provide subscription plans to individuals, groups, or businesses who use social media platforms professionally. Hootsuite differs from TweetDeck in that it allows users to monitor all social media applications at once and gives users the ability to prepare social media releases in advance of specific campaigns or events. Using these applications, conference seminars and events can be posted from multiple Twitter or mixed social media platforms at once.

## CONFERENCE COMMUNICATIONS: CAREER OR *AD HOC*?

In academic science, Twitter and other social media platforms have evolved from tools of personal communication to tools by which networking, public engagement, and scientific discussion take place (18–22). At present, live-tweeting of scientific conferences is often undertaken by a mix of conference organizers and graduate or postdoctoral researchers who do so freely out of interest, note-taking, or to generate discussion. In many cases, learned societies and institutes co-opt a student or postdoctoral member with more social media savvy to live-tweet conferences for their scientific community. While this practice can increase a junior scientist’s recognition and online following, it can also become time-consuming, with few funding agencies rewarding early-career scientists (ECRs) for time spent outside of primary research.

With the advent of alternative metrics (“altmetrics”) as a measure of scientific research impact, services to science communication, such as live-tweeting scientific conferences, may be recognized as a valuable contribution to education and the democratization of scientific information, perhaps being incorporated into portfolios for academic promotion and tenure (23–25). Until this practice becomes commonplace, it may be best if the organizers of scientific conferences implement live-tweeting as part of their communication strategies to provide maximum outreach while ensuring that unpaid volunteers from academia are not overworked or taken advantage of.

At present, Twitter remains the primary social media platform for real-time scientific conference updates. Even so, as the landscape of communications technology and software applications changes over time, so too will the communication of science and the avenues by which scientists network and engage with the general public. As the COVID-19 pandemic made conferences and work more virtual in nature, applications such as Zoom (https://zoom.us) and Slack (https://slack.com) have gained favor within the research community. In the future, conference notifications and science communication may move to new apps, such as Reddit (https://www.reddit.com), Discord (https://discord.com), Mastodon (https://joinmastodon.org), or others, and science communicators will need to move with them.
